# Supplementary material for: A population-based cohort study of longitudinal change of high-density lipoprotein cholesterol impact on gastrointestinal cancer risk
Source: Nat Commun. 2024 Apr 4;15:2923. doi: 10.1038/s41467-024-47193-9 (PMC10994902; doi:10.1038/s41467-024-47193-9)
Supplement: Supplementary file 1 — Supplementary Information [file 41467_2024_47193_MOESM1_ESM.pdf]

Supplementary Information

Impact of Longitudinal Change of High-density Lipoprotein Cholesterol on Human Gastrointestinal Cancer Risk

Supplementary Table 1. Baseline characteristics of included and excluded person

Supplementary Table 2. Gastrointestinal cancer risk by baseline HDL-C

Supplementary Table 3. Sensitivity analysis I (Model II)

Supplementary Table 4. Sensitivity analysis II (Model III)

Supplementary Table 5. Interaction analysis

Supplementary Table 6. Subgroup analysis by combination of sex and smoking status

Supplementary Table 7. Gastrointestinal cancer risk by further categorization of HDL-C change

Supplementary Table 8. Gastrointestinal cancer risk by absolute HDL-C change

Supplementary Table 9. Comparison of HDL cholesterol-cancer association with those from current study and literature review

Supplementary Table 1. Baseline characteristics of included and excluded person

	Included person (n=3,130,795)	Excluded person[‡] (n=2,969,620)
Age, year, median (IQR)	54 (62 - 46)	51 (60 - 44)
Economic status, median (IQR)*	13 (17 - 8)	12 (17 - 5)
BMI, kg/m2, median (IQR)	23 (21.9 - 25.8)	23.6 (25.7 - 21.6)
HDL-C, mg/dL, median (IQR)	53 (63 - 45)	54 (63 - 46)
Men, no (%)	1,387,648 (44.3)	1,260,926 (42.6)
Hypertension, no (%)	1,007,102 (32.2)	837,396 (28.2)
Heart disease, no (%)	119,544 (3.8)	97,420 (3.3)
Cerebrovascular disease, no (%)	64,282 (2.1)	53,835 (1.8)
Diabetes mellitus, no (%)	344,913 (11)	303,893 (10.2)
Lipid lowering drug, no (%)	116,404 (3.7)	85,215 (2.9)
Drinking status, no (%)		
None	1,905,220 (61)	1,757,993 (59.4)
1/week	493,854 (15.8)	518,934 (17.5)
2-3/week	509,416 (16.3)	499,883 (16.9)
4-5/week	125,588 (4)	111,604 (3.8)
≥6/week	88,065 (2.8)	73,141 (2.5)
Smoking status, no (%)		
Never	2,124,822 (68.1)	2,015,960 (68.1)
Past	481,076 (15.4)	428,112 (14.5)
Current	516,356 (16.5)	517,332 (17.5)
Moderate activity, no (%)[†]		
None	1,793,307 (57.5)	1,683,826 (56.9)
1-2 day/week	656,980 (21.1)	677,887 (22.9)

3-5 day/week	510,267 (16.4)	466,660 (15.8)
6-7 day/week	160,249 (5.1)	131,885 (4.5)

BMI, body mass index; HDL-C, high density lipoprotein; SD, standard deviation.

*Economic status is twentile (1-20). 1 is the lowest and 20 is the highest income.

†Moderate physical activity refers to “walking or exercising and feeling mild dyspnea for more than 30 min per day.”

‡Excluded person among all persons who underwent baseline NGHE. This group includes persons who did not undergo national cancer screening and individuals who expired or developed any cancer within 1 years after baseline enrollment.

Supplementary Table 2. Gastrointestinal cancer risk by baseline HDL-C

		Total		Men		Women	
	**Binary HDL-C**	**HR (95% CI) ***	**P-value**	**HR (95% CI) ***	**P-value**	**HR (95% CI) ***	**P-value**
**Gastric cancer (C16)**	Low HDL-C	1		1		1	
	Normal HDL-C	0.96 (0.93-0.98)	0.001	0.98 (0.94-1.01)	0.170	0.98 (0.95-1.02)	0.300
**Colorectal cancer (C18-C20)**	Low HDL-C	1		1		1	
	Normal HDL-C	0.98 (0.95-1.00)	0.090	1.00 (0.96-1.04)	0.980	0.95 (0.92-0.98)	0.002
**Liver cancer (C22) †**	Low HDL-C	1		1		1	
	Normal HDL-C	0.98 (0.95-1.02)	0.400	0.93 (0.88-0.97)	0.002	1.07 (1.00-1.14)	0.040
**Pancreatic (C25)**	Low HDL-C	1		1		1	
	Normal HDL-C	0.92 (0.88-0.97)	<0.001	0.92 (0.85-0.99)	0.022	0.91 (0.85-0.97)	0.004
**GB/biliary (c23, C24)**	Low HDL-C	1		1		1	
	Normal HDL-C	0.99 (0.94-1.04)	0.690	1.00 (0.93-1.08)	0.939	0.98 (0.92-1.05)	0.676

*Adjusted for age, sex, body mass index, hypertension, diabetes, cerebrovascular disease, heart disease, smoking status, drinking status, LDL, TG, lipid lowering drug, and physical activity.

†Liver factors (chronic hepatitis B, chronic hepatitis C, and liver cirrhosis) were additionally adjusted in liver cancer analysis. CI, confidence interval; HDL-C, high density lipoprotein cholesterol; HR, hazard ratio.

Low HDL-C refers to HDL-C < 40mg/dL in men and < 50mg/dL in women. Normal HDL-C means HDL-C ≥ 40mg/dL in men and ≥ 50mg/dL in women.

Supplementary Table 3. Sensitivity analysis I (Model II)

	HDL-C change	Number of case	Unadjusted HR		Adjusted HR*		Adjusted HR**	
			HR (95% CI)	p-value	HR (95% CI)	p-value	HR (95% CI)	p-value
**Any cancer**	Low → Low	13,367	1.02 (1.01, 1.04)	0.01	1.06 (1.04, 1.08)	<.0001	1.06 (1.04, 1.09)	<.0001
	Low → Normal	10,658	0.98 (0.96, 1)	0.05	1.01 (0.99, 1.03)	0.35	1.02 (1, 1.04)	0.12
	Normal → Low	14,444	1.13 (1.11, 1.15)	<.0001	1.1 (1.08, 1.12)	<.0001	1.1 (1.08, 1.12)	<.0001
	Normal→ Normal	67,306	1		1		1	
**Gastric cancer**	Low → Low	4,501	0.93 (0.9, 0.96)	<.0001	1.08 (1.04, 1.11)	<.0001	1.06 (1.03, 1.1)	0.00
	Low → Normal	3,775	0.94 (0.91, 0.97)	<0.001	1.03 (1, 1.07)	0.09	1.02 (0.99, 1.06)	0.22
	Normal → Low	4,896	1.04 (1.01, 1.07)	0.02	1.07 (1.03, 1.1)	<.0001	1.06 (1.03, 1.1)	<0.001
	Normal→ Normal	24,973	1		1		1	
**Colorectal cancer**	Low → Low	4,304	1.07 (1.04, 1.11)	<.0001	1.05 (1.01, 1.08)	0.01	1.02 (0.98, 1.06)	0.31
	Low → Normal	3,275	0.98 (0.94, 1.02)	0.24	0.98 (0.94, 1.02)	0.24	0.96 (0.92, 1)	0.03
	Normal → Low	4,548	1.16 (1.13, 1.2)	<.0001	1.1 (1.06, 1.13)	<.0001	1.09 (1.05, 1.12)	<.0001
	Normal→ Normal	20,695	1		1		1	
**Liver cancer**†	Low → Low	2469	0.99 (0.95, 1.03)	0.64	1.07 (1.02, 1.12)	0.01	1.17 (1.11, 1.23)	<.0001
	Low → Normal	1942	0.93 (0.89, 0.98)	0.004	0.99 (0.94, 1.04)	0.73	1.07 (1.02, 1.13)	0.01
	Normal → Low	2975	1.22 (1.18, 1.27)	<.0001	1.21 (1.16, 1.26)	<.0001	1.26 (1.2, 1.31)	<.0001
	Normal→ Normal	12,865	1		1		1	
**Pancreatic cancer**	Low → Low	1597	1.22 (1.15, 1.29)	<.0001	1.04 (0.99, 1.11)	0.15	1.05 (0.99, 1.11)	0.12
	Low → Normal	1240	1.13 (1.07, 1.2)	<.0001	1.04 (0.97, 1.1)	0.28	1.04 (0.98, 1.11)	0.24
	Normal → Low	1603	1.26 (1.19, 1.33)	<.0001	1.1 (1.04, 1.16)	0.002	1.1 (1.04, 1.16)	0.001
	Normal→ Normal	6,751	1		1		1	
**Gallbladder cancer**	Low → Low	563	1.17 (1.07, 1.29)	0.001	0.96 (0.87, 1.06)	0.43	0.95 (0.86, 1.05)	0.34
	Low → Normal	447	1.12 (1.01, 1.24)	0.03	0.99 (0.89, 1.1)	0.83	0.98 (0.88, 1.09)	0.74
	Normal → Low	606	1.3 (1.19, 1.42)	<.0001	1.1 (1.01, 1.21)	0.04	1.1 (1, 1.21)	0.05

	Normal→ Normal	2468	1		1		1	
**Biliary cancer**	Low → Low	869	1.02 (0.95, 1.1)	0.53	0.94 (0.87, 1.02)	0.13	0.95 (0.88, 1.03)	0.19
	Low → Normal	686	0.97 (0.89, 1.05)	0.42	0.95 (0.87, 1.03)	0.18	0.95 (0.87, 1.03)	0.24
	Normal → Low	929	1.12 (1.05, 1.21)	0.001	1.02 (0.95, 1.1)	0.53	1.03 (0.95, 1.11)	0.49
	Normal→ Normal	4,375	1		1		1	

[*]Adjusted for age, sex, economic status, body mass index, hypertension, diabetes, cerebrovascular disease, heart disease, smoking status, drinking status, physical activity, and use of lipid lowering drug.

[†]Adjusted for age, sex, economic status, body mass index, hypertension, diabetes, cerebrovascular disease, heart disease, smoking status, drinking status, physical activity, use of lipid lowering drug, liver factors (chronic liver disease, chronic hepatitis B, chronic hepatitis C, and liver cirrhosis) and triglyceride.

[**]Adjusted for age, sex, economic status, body mass index, hypertension, diabetes, cerebrovascular disease, heart disease, smoking status, drinking status, physical activity, LDL, TG, and use of lipid lowering drug.

CI, confidence interval; HDL-C, high density lipoprotein cholesterol; HR, hazard ratio. Low HDL-C refers to <40mg/dL in men and <50mg/dL in women. Normal HDL-C means ≥40mg/dL in men and ≥50mg/dL in women.

Exclusion any cancer within 2 years from baseline.

Adjusted HRs and CIs are derived from Cox proportional regression analysis. All statistical tests are two-sided.

Supplementary Table 4. Sensitivity analysis II (Model III)

	HDL-C change	Number of case	Unadjusted HR		Adjusted HR*		Adjusted HR**	
			HR (95% CI)	p-value	HR (95% CI)	p-value	HR (95% CI)	p-value
**Gastric cancer**	Low → Low	3,250	0.93 (0.89, 0.96)	<0.001	1.07 (1.03, 1.12)	<0.001	1.06 (1.02, 1.1)	<0.001
	Low → Normal	2,638	0.9 (0.86, 0.94)	<0.001	0.99 (0.95, 1.04)	0.74	0.98 (0.94, 1.03)	0.47
	Normal → Low	3,496	1.02 (0.98, 1.06)	0.27	1.06 (1.02, 1.1)	<0.001	1.05 (1.01, 1.09)	0.01
	Normal→ Normal	18,152	1		1		1	
**Colorectal cancer**	Low → Low	3,215	1.08 (1.04, 1.12)	<0.001	1.04 (1, 1.09)	0.04	1.02 (0.98, 1.06)	0.33
	Low → Normal	2,429	0.98 (0.94, 1.02)	0.29	0.98 (0.94, 1.02)	0.33	0.96 (0.92, 1.01)	0.09
	Normal → Low	3,307	1.14 (1.1, 1.18)	<0.001	1.08 (1.04, 1.12)	<0.001	1.07 (1.03, 1.11)	<0.001
	Normal→ Normal	15,345	1		1		1	
**Liver cancer**[†]	Low → Low	1995	0.98 (0.94, 1.03)	0.49	1.06 (1, 1.11)	0.04	1.15 (1.09, 1.21)	<0.001
	Low → Normal	1600	0.94 (0.9, 1)	0.03	1 (0.94, 1.06)	0.96	1.07 (1.01, 1.14)	0.01
	Normal → Low	2297	1.16 (1.11, 1.22)	<0.001	1.14 (1.09, 1.2)	<0.001	1.18 (1.13, 1.24)	<0.001
	Normal→ Normal	10,461	1		1		1	
**Pancreatic cancer**	Low → Low	1382	1.2 (1.14, 1.28)	<0.001	1.02 (0.96, 1.09)	0.55	1.02 (0.96, 1.09)	0.48
	Low → Normal	1091	1.14 (1.07, 1.21)	<0.001	1.03 (0.96, 1.1)	0.42	1.03 (0.96, 1.1)	0.38
	Normal → Low	1353	1.21 (1.14, 1.28)	<0.001	1.05 (0.99, 1.12)	0.1	1.06 (0.99, 1.12)	0.09
	Normal→ Normal	5,914	1		1		1	
**Gallbladder cancer**	Low → Low	471	1.16 (1.05, 1.28)	<0.01	0.95 (0.85, 1.05)	0.3	0.94 (0.84, 1.05)	0.24
	Low → Normal	384	1.13 (1.01, 1.26)	0.03	1.01 (0.9, 1.13)	0.89	1 (0.89, 1.12)	0.97
	Normal → Low	497	1.25 (1.14, 1.38)	<0.01	1.07 (0.96, 1.18)	0.22	1.06 (0.96, 1.18)	0.24
	Normal→ Normal	2095	1		1		1	
**Biliary cancer**	Low → Low	735	1 (0.93, 1.08)	0.99	0.92 (0.84, 1)	0.04	0.93 (0.85, 1.01)	0.09
	Low → Normal	582	0.95 (0.87, 1.04)	0.23	0.92 (0.84, 1.01)	0.09	0.94 (0.85, 1.03)	0.15
	Normal → Low	754	1.05 (0.98, 1.14)	0.19	0.96 (0.89, 1.04)	0.31	0.97 (0.89, 1.05)	0.39

Normal→ Normal	3,783	1	1	1

*Adjusted for age, sex, economic status, body mass index, hypertension, diabetes, cerebrovascular disease, heart disease, smoking status, drinking status, physical activity, and use of lipid lowering drug.

†Adjusted for age, sex, economic status, body mass index, hypertension, diabetes, cerebrovascular disease, heart disease, smoking status, drinking status, physical activity, use of lipid lowering drug, liver factors (chronic liver disease, chronic hepatitis B, chronic hepatitis C, and liver cirrhosis) and triglyceride.

**Adjusted for age, sex, economic status, body mass index, hypertension, diabetes, cerebrovascular disease, heart disease, smoking status, drinking status, physical activity, LDL, TG, and use of lipid lowering drug.

CI, confidence interval; HDL-C, high density lipoprotein cholesterol; HR, hazard ratio. Low HDL-C refers to <40mg/dL in men and <50mg/dL in women. Normal HDL-C means ≥40mg/dL in men and ≥50mg/dL in women.

Exclusion any cancers up to 2nd measurement of HDL-C

Adjusted HRs and CIs are derived from Cox proportional regression analysis. All statistical tests are two-sided.

Supplementary Table 5. Interaction analysis (Joint test)

Interaction between sex and HDL-C change in the cancer risk				Interaction between smoking and HDL-C change in the cancer risk				Interaction between age group and HDL-C change in the cancer risk			
**Effect**	**DF**	**Wald Chi-Square**	**P-value**	**Effect**	**DF**	**Wald Chi-Square**	**P-value**	**Effect**	**DF**	**Wald Chi-Square**	**P-value**
**Gastric cancer**											
HDL_C	3	264.17	<.0001	HDL_C	3	11.5726	0.009	HDL_C	3	272.5719	<.0001
SEX_TYPE	1	5809.386	<.0001	Q_SMK_YN	2	2936.877	<.0001	AGE_CAT_60	1	9146.274	<.0001
HDL_C*SEX_TYPE	3	64.7169	<.0001	HDL_C*Q_SMK_YN	6	18.0888	0.006	HDL_C*AGE_CAT_60	3	42.2341	<.0001
**Liver cancer**											
HDL_C	3	214.3463	<.0001	HDL_C	3	101.483	<.0001	HDL_C	3	90.1842	<.0001
SEX_TYPE	1	3118.326	<.0001	Q_SMK_YN	2	1435.403	<.0001	AGE_CAT_60	1	4113.162	<.0001
HDL_C*SEX_TYPE	3	17.0693	0.0007	HDL_C*Q_SMK_YN	6	39.4402	<.0001	HDL_C*AGE_CAT_60	3	34.3295	<.0001
**Colorectal cancer**											
HDL_C	3	265.9314	<.0001	HDL_C	3	17.4558	0.0006	HDL_C	3	96.1581	<.0001
SEX_TYPE	1	1948.034	<.0001	Q_SMK_YN	2	876.8675	<.0001	AGE_CAT_60	1	7521.474	<.0001
HDL_C*SEX_TYPE	3	43.4193	<.0001	HDL_C*Q_SMK_YN	6	22.683	0.0009	HDL_C*AGE_CAT_60	3	46.0177	<.0001
**Pancreatic cancer**											
HDL_C	3	154.9761	<.0001	HDL_C	3	30.8831	<.0001	HDL_C	3	2.411	0.4916
SEX_TYPE	1	202.3745	<.0001	Q_SMK_YN	2	52.6888	<.0001	AGE_CAT_60	1	2869.315	<.0001
HDL_C*SEX_TYPE	3	20.3697	0.0001	HDL_C*Q_SMK_YN	6	11.3824	0.0773	HDL_C*AGE_CAT_60	3	27.5208	<.0001
**Gallbladder cancer**											
HDL_C	3	58.9592	<.0001	HDL_C	3	19.2718	0.0002	HDL_C	3	11.41	0.0097
SEX_TYPE	1	60.9395	<.0001	Q_SMK_YN	2	15.4708	0.0004	AGE_CAT_60	1	1543.302	<.0001
HDL_C*SEX_TYPE	3	24.3317	<.0001	HDL_C*Q_SMK_YN	6	11.9914	0.0622	HDL_C*AGE_CAT_60	3	16.8603	0.0008
**Biliary cancer**											
HDL_C	3	70.9104	<.0001	HDL_C	3	3.6549	0.3012	HDL_C	3	45.4559	<.0001
SEX_TYPE	1	540.1425	<.0001	Q_SMK_YN	2	137.6	<.0001	AGE_CAT_60	1	2883.836	<.0001
HDL_C*SEX_TYPE	3	18.6374	0.0003	HDL_C*Q_SMK_YN	6	1.3017	0.9716	HDL_C*AGE_CAT_60	3	22.8711	<.0001

DF, degree of freedom; HDL_C, HDL-C change group (4 groups); SMK, smoking status (never, past, current smokers).

Supplementary Table 6. Subgroup analysis by combination of sex and smoking status

		men-smoker		men-never smoker		women-smoker		women-never smoker	
	HDL-C change	HR (95% CI) *	P value	HR (95% CI) *	P value	HR (95% CI) *	P value	HR (95% CI) *	P value
**Gastric cancer**	Low → Low	1.08 (1.02, 1.14)	0.014	0.97 (0.89, 1.06)	0.463	1.01 (0.79, 1.3)	0.926	1.11 (1.06, 1.17)	<.0001
	Low → Normal	1.02 (0.97, 1.08)	0.409	1.01 (0.93, 1.09)	0.910	1.15 (0.88, 1.5)	0.299	1.07 (1.01, 1.13)	0.023
	Normal → Low	1.06 (1.01, 1.11)	0.015	1.06 (0.99, 1.14)	0.101	1.19 (0.93, 1.52)	0.175	1.07 (1.01, 1.12)	0.015
	Normal→ Normal	1		1		1		1	
**Liver cancer**†	Low → Low	1.23 (1.13, 1.33)	<.0001	1.07 (0.95, 1.21)	0.246	1.6 (1.15, 2.23)	0.005	1.2 (1.12, 1.29)	<.0001
	Low → Normal	1.09 (1.01, 1.18)	0.034	1.09 (0.98, 1.22)	0.119	1.46 (1.01, 2.1)	0.045	1.04 (0.96, 1.13)	0.318
	Normal → Low	1.37 (1.28, 1.45)	<.0001	1.21 (1.1, 1.33)	<.0001	1.23 (0.85, 1.76)	0.271	1.23 (1.14, 1.32)	<.0001
	Normal→ Normal	1		1		1		1	
**Colorectal cancer**	Low → Low	1.03 (0.96, 1.1)	0.409	1.01 (0.92, 1.11)	0.850	0.93 (0.74, 1.16)	0.497	1.05 (1, 1.1)	0.048
	Low → Normal	0.95 (0.89, 1.02)	0.134	0.97 (0.88, 1.06)	0.473	0.95 (0.74, 1.21)	0.679	0.99 (0.94, 1.04)	0.711
	Normal → Low	1.11 (1.05, 1.17)	0.000	1.09 (1.01, 1.18)	0.026	1.03 (0.83, 1.29)	0.782	1.09 (1.04, 1.14)	0.001
	Normal→ Normal	1		1		1		1	
**Pancreatic cancer**	Low → Low	1.03 (0.9, 1.18)	0.664	1.01 (0.84, 1.21)	0.936	0.92 (0.64, 1.34)	0.677	1.08 (1.01, 1.17)	0.034
	Low → Normal	1.22 (1.09, 1.37)	0.001	1 (0.84, 1.19)	0.962	0.81 (0.52, 1.26)	0.349	1 (0.92, 1.09)	0.993
	Normal → Low	1.15 (1.03, 1.27)	0.010	1.09 (0.95, 1.26)	0.231	1.01 (0.69, 1.47)	0.972	1.12 (1.04, 1.21)	0.003
	Normal→ Normal	1		1		1		1	
**Gallbladder cancer**	Low → Low	0.81 (0.63, 1.03)	0.088	0.74 (0.53, 1.03)	0.078	0.99 (0.53, 1.88)	0.986	1.04 (0.93, 1.18)	0.487
	Low → Normal	1.04 (0.85, 1.28)	0.705	0.94 (0.71, 1.24)	0.655	1.2 (0.61, 2.37)	0.604	0.99 (0.87, 1.14)	0.928
	Normal → Low	1.32 (1.12, 1.56)	0.001	1.15 (0.92, 1.43)	0.216	1.36 (0.73, 2.54)	0.340	1.03 (0.91, 1.17)	0.626
	Normal→ Normal	1		1		1		1	
**Biliary cancer**	Low → Low	1.01 (0.86, 1.18)	0.944	0.86 (0.69, 1.07)	0.182	0.71 (0.39, 1.27)	0.243	0.99 (0.89, 1.09)	0.777
	Low → Normal	0.96 (0.82, 1.11)	0.574	1.03 (0.85, 1.25)	0.750	1.48 (0.89, 2.46)	0.135	0.92 (0.81, 1.04)	0.163
	Normal → Low	0.99 (0.87, 1.12)	0.856	1.03 (0.88, 1.21)	0.720	1.68 (1.06, 2.67)	0.027	1.07 (0.96, 1.19)	0.217

Normal→ Normal	1	1	1	1

*Adjusted for sex, economic status, body mass index, hypertension, diabetes, cerebrovascular disease, heart disease, SMK, drinking status, physical activity, LDL, TG, and use of lipid lowering drug.

†Adjusted for sex, economic status, body mass index, hypertension, diabetes, cerebrovascular disease, heart disease, SMK, drinking status, physical activity, LDL, TG, use of lipid lowering drug, liver factors (chronic liver disease, chronic hepatitis B, chronic hepatitis C, and liver cirrhosis) and triglyceride.

Adjusted HRs and CIs are derived from Cox proportional regression analysis. All statistical tests are two-sided.

Supplementary Table 7. Gastrointestinal cancer risk by further categorization of HDL-C change

	HDL-C change	Total number	Number of cancer	Unadjusted HR		Adjusted HR*	
				HR (95% CI)	p-value	HR (95% CI)	p-value
**Gastric cancer**	**Low-Increase**	125,709	1,525	0.92 (0.88, 0.97)	0.002	1.05 (1.00, 1.11)	0.055
	**Low-Low**	387,739	4,717	0.93 (0.90, 0.96)	<.0001	1.06 (1.03, 1.10)	0.001
	**Low-Normal**	205,486	2,583	0.95 (0.92, 0.99)	0.022	1.02 (0.98, 1.06)	0.388
	**Normal-Increase**	194,998	2,555	0.99 (0.95, 1.03)	0.73	1.03 (0.99, 1.08)	0.153
	**Normal-Low**	385,774	5,178	1.03 (1.00, 1.07)	0.031	1.06 (1.03, 1.09)	0
	**Normal-Normal**	1,831,089	24,138	1		1	
**Colorectal cancer**	**Low-Increase**	125,709	1,356	0.96 (0.91, 1.02)	0.149	0.95 (0.90, 1.01)	0.08
	**Low-Low**	387,739	4,594	1.06 (1.03, 1.10)	0	1.02 (0.98, 1.05)	0.356
	**Low-Normal**	205,486	2,269	0.98 (0.94, 1.03)	0.444	0.97 (0.93, 1.01)	0.146
	**Normal-Increase**	194,998	2,032	0.93 (0.89, 0.97)	0.001	0.94 (0.90, 0.99)	0.013
	**Normal-Low**	385,774	4,919	1.15 (1.12, 1.19)	<.0001	1.08 (1.05, 1.12)	<.0001
	**Normal-Normal**	1,831,089	20,537	1		1	
**Liver cancer**†	**Low-Increase**	125,709	886	1.06 (0.99, 1.13)	0.104	1.31 (1.22, 1.41)	<.0001
	**Low-Low**	387,739	2,550	1.00 (0.95, 1.04)	0.858	1.20 (1.14, 1.26)	<.0001
	**Low-Normal**	205,486	1,184	0.87 (0.82, 0.92)	<.0001	0.97 (0.91, 1.04)	0.383
	**Normal-Increase**	194,998	1,386	1.07 (1.01, 1.13)	0.022	1.15 (1.08, 1.21)	<.0001
	**Normal-Low**	385,774	3,138	1.24 (1.19, 1.29)	<.0001	1.29 (1.24, 1.35)	<.0001
	**Normal-Normal**	1,831,089	12,165	1		1	
**Pancreatic cancer**	**Low-Increase**	125,709	516	1.19 (1.09, 1.30)	0	1.07 (0.98, 1.18)	0.143
	**Low-Low**	387,739	1,621	1.22 (1.16, 1.29)	<.0001	1.05 (0.99, 1.12)	0.082
	**Low-Normal**	205,486	784	1.11 (1.03, 1.19)	0.008	1.03 (0.95, 1.11)	0.473
	**Normal-Increase**	194,998	657	0.98 (0.90, 1.06)	0.573	1.00 (0.92, 1.08)	0.909

	**Normal-Low**	385,774	1,660	1.27 (1.20, 1.34)	<.0001	1.11 (1.05, 1.18)	0
	**Normal-Normal**	1,831,089	6,294	1		1	
**Gallbladder cancer**	**Low-Increase**	125,709	199	1.25 (1.08, 1.45)	0.003	1.06 (0.91, 1.23)	0.481
	**Low-Low**	387,739	582	1.20 (1.09, 1.31)	0	0.97 (0.88, 1.07)	0.561
	**Low-Normal**	205,486	271	1.04 (0.92, 1.18)	0.505	0.95 (0.83, 1.08)	0.402
	**Normal-Increase**	194,998	234	0.95 (0.83, 1.09)	0.454	0.96 (0.84, 1.11)	0.586
	**Normal-Low**	385,774	634	1.32 (1.21, 1.44)	<.0001	1.12 (1.02, 1.23)	0.013
	**Normal-Normal**	1,831,089	2,305	1		1	
**Biliary cancer**	**Low-Increase**	125,709	245	0.88 (0.77, 1.00)	0.044	0.87 (0.76, 0.99)	0.033
	**Low-Low**	387,739	881	1.03 (0.96, 1.11)	0.418	0.96 (0.88, 1.03)	0.254
	**Low-Normal**	205,486	469	1.03 (0.93, 1.13)	0.591	1.01 (0.91, 1.12)	0.852
	**Normal-Increase**	194,998	437	1.01 (0.91, 1.11)	0.874	1.04 (0.94, 1.15)	0.408
	**Normal-Low**	385,774	961	1.14 (1.06, 1.22)	0	1.04 (0.97, 1.12)	0.278
	**Normal-Normal**	1,831,089	4,058	1		1	

*Adjusted for age, sex, economic status, body mass index, hypertension, diabetes, cerebrovascular disease, heart disease, smoking status, drinking status, physical activity, LDL, TG, and use of lipid lowering drug.

†Adjusted for age, sex, economic status, body mass index, hypertension, diabetes, cerebrovascular disease, heart disease, smoking status, drinking status, physical activity, LDL, TG, use of lipid lowering drug, and liver factors (chronic liver disease, chronic hepatitis B, chronic hepatitis C, and liver cirrhosis).

CI, confidence interval; HDL-C, high density lipoprotein cholesterol; HR, hazard ratio. Low HDL-C refers to <40mg/dL in men and <50mg/dL in women. Normal HDL-C means ≥40mg/dL in men and ≥50mg/dL in women.

Increase means ΔHDL-C [(HDL-C at follow-up) - (HDL-C baseline)] ≥ 15mg/dL. Detail definition was provided in Fig 1C and Method.

Adjusted HRs and CIs are derived from Cox proportional regression analysis. All statistical tests are two-sided.

Supplementary Table 8. Gastrointestinal cancer risk by absolute HDL-C change

	HDL-C change	Total number	Number of cancer	Unadjusted HR		Adjusted HR*		Adjusted HR**	
				HR (95% CI)	p-value	HR (95% CI)	p-value	HR (95% CI)	p-value
**Gastric cancer**	**ΔHDL<-10**	561153	7,747	1.1 (1.07, 1.13)	<.0001	1.05 (1.02, 1.08)	0.003	1.05 (1.02, 1.08)	0.002
	**ΔHDL: -10~ -5**	424815	5,573	1.03 (1, 1.06)	0.051	1.02 (0.99, 1.05)	0.273	1.02 (0.99, 1.05)	0.236
	**ΔHDL: -5~5**	1121127	14,327	1		1		1	
	**ΔHDL: 5-15**	702993	8,969	1 (0.97, 1.02)	0.745	1.03 (1, 1.05)	0.077	1.02 (1, 1.05)	0.093
	**ΔHDL:15-25**	234723	2,944	0.98 (0.94, 1.02)	0.318	1.03 (0.99, 1.08)	0.130	1.03 (0.99, 1.07)	0.147
	**ΔHDL≥25**	85984	1,136	1.04 (0.98, 1.1)	0.239	1.07 (1, 1.14)	0.036	1.07 (1, 1.14)	0.038
**Colorectal cancer**	**ΔHDL<-10**	561153	7,143	1.14 (1.11, 1.18)	<.0001	1.07 (1.04, 1.1)	<.0001	1.07 (1.04, 1.11)	<.0001
	**ΔHDL: -10~ -5**	424815	4,978	1.04 (1.01, 1.08)	0.013	1.03 (0.99, 1.06)	0.146	1.03 (0.99, 1.06)	0.106
	**ΔHDL: -5~5**	1121127	12,663	1		1		1	
	**ΔHDL: 5-15**	702993	7,535	0.95 (0.92, 0.97)	0.000	0.96 (0.93, 0.99)	0.008	0.96 (0.93, 0.99)	0.006
	**ΔHDL:15-25**	234723	2,447	0.92 (0.88, 0.96)	0.000	0.94 (0.9, 0.98)	0.004	0.94 (0.9, 0.98)	0.003
	**ΔHDL≥25**	85984	941	0.97 (0.91, 1.04)	0.370	0.97 (0.91, 1.04)	0.357	0.97 (0.9, 1.03)	0.322
**Liver cancer†**	**ΔHDL<-10**	561153	4,829	1.39 (1.34, 1.44)	<.0001	1.32 (1.27, 1.37)	<.0001	1.28 (1.24, 1.34)	<.0001
	**ΔHDL: -10~ -5**	424815	2,831	1.06 (1.02, 1.11)	0.006	1.05 (1, 1.1)	0.051	1.04 (0.99, 1.09)	0.113
	**ΔHDL: -5~5**	1121127	7,065	1		1		1	
	**ΔHDL: 5-15**	702993	4,312	0.97 (0.93, 1.01)	0.112	1.01 (0.97, 1.05)	0.805	1.01 (0.97, 1.05)	0.601
	**ΔHDL:15-25**	234723	1,558	1.05 (0.99, 1.11)	0.083	1.13 (1.07, 1.2)	<.0001	1.14 (1.07, 1.21)	<.0001
	**ΔHDL≥25**	85984	714	1.32 (1.22, 1.42)	<.0001	1.39 (1.28, 1.51)	<.0001	1.41 (1.3, 1.53)	<.0001
	**ΔHDL<-10**	561153	2,298	1.16 (1.1, 1.22)	<.0001	1.07 (1.02, 1.13)	0.009	1.07 (1.02, 1.13)	0.009

**Pancreatic cancer**	**ΔHDL: -10~ -5**	424815	1,576	1.04 (0.98, 1.1)	0.171	1.01 (0.95, 1.07)	0.741	1.01 (0.95, 1.07)	0.750
	**ΔHDL: -5~5**	1121127	4,015	1		1		1	
	**ΔHDL: 5-15**	702993	2,470	0.98 (0.93, 1.03)	0.363	0.98 (0.93, 1.04)	0.523	0.98 (0.94, 1.04)	0.530
	**ΔHDL:15-25**	234723	850	1.01 (0.94, 1.08)	0.853	1 (0.93, 1.08)	0.967	1 (0.93, 1.08)	0.973
	**ΔHDL≥25**	85984	323	1.05 (0.94, 1.18)	0.405	1.05 (0.94, 1.18)	0.405	1.05 (0.94, 1.18)	0.403
**Gallbladder cancer**	**ΔHDL<-10**	561153	862	1.21 (1.11, 1.31)	<.0001	1.1 (1.01, 1.2)	0.028	1.1 (1.01, 1.2)	0.027
	**ΔHDL: -10~ -5**	424815	588	1.07 (0.98, 1.18)	0.146	1.04 (0.94, 1.15)	0.416	1.04 (0.95, 1.15)	0.404
	**ΔHDL: -5~5**	1121127	1,453	1		1		1	
	**ΔHDL: 5-15**	702993	889	0.97 (0.89, 1.06)	0.495	0.99 (0.91, 1.08)	0.767	0.99 (0.91, 1.07)	0.748
	**ΔHDL:15-25**	234723	305	1 (0.88, 1.13)	0.972	0.98 (0.86, 1.11)	0.733	0.98 (0.86, 1.11)	0.718
	**ΔHDL≥25**	85984	128	1.15 (0.96, 1.38)	0.135	1.13 (0.94, 1.36)	0.199	1.13 (0.94, 1.36)	0.199
**Biliary cancer**	**ΔHDL<-10**	561153	1,422	1.18 (1.1, 1.26)	<.0001	1.1 (1.02, 1.17)	0.008	1.09 (1.02, 1.17)	0.010
	**ΔHDL: -10~ -5**	424815	907	0.98 (0.91, 1.06)	0.632	0.97 (0.89, 1.05)	0.391	0.97 (0.89, 1.04)	0.372
	**ΔHDL: -5~5**	1121127	2,452	1		1		1	
	**ΔHDL: 5-15**	702993	1,588	1.03 (0.97, 1.1)	0.385	1.06 (0.99, 1.13)	0.080	1.06 (0.99, 1.13)	0.076
	**ΔHDL:15-25**	234723	485	0.94 (0.85, 1.04)	0.220	0.97 (0.88, 1.07)	0.509	0.97 (0.88, 1.07)	0.520
	**ΔHDL≥25**	85984	197	1.05 (0.91, 1.21)	0.528	1.08 (0.94, 1.26)	0.283	1.09 (0.94, 1.26)	0.270

Absolute HDL change [(follow-up HDL-C) - (baseline HDL-C)], mg/dL

*Adjusted for age, sex, economic status, body mass index, hypertension, diabetes, cerebrovascular disease, heart disease, smoking status, drinking status, physical activity, and use of lipid lowering drug.

†Adjusted for age, sex, economic status, body mass index, hypertension, diabetes, cerebrovascular disease, heart disease, smoking status, drinking status, physical activity, use of lipid lowering drug, LDL, TG, and liver factors (chronic liver disease, chronic hepatitis B, chronic hepatitis C, and liver cirrhosis).

**Adjusted for age, sex, economic status, body mass index, hypertension, diabetes, cerebrovascular disease, heart disease, smoking status, drinking status, physical activity,

LDL, TG, and use of lipid lowering drug.

CI, confidence interval; HDL-C, high density lipoprotein cholesterol; HR, hazard ratio. Low HDL-C refers to <40mg/dL in men and <50mg/dL in women. Normal HDL-C means ≥40mg/dL in men and ≥50mg/dL in women.

Adjusted HRs and CIs are derived from Cox proportional regression analysis. All statistical tests are two-sided.

Supplementary Table 9. Comparison of HDL cholesterol-cancer association with those from current study and literature review

Cancer site (ICD10) 1st author year	Design	Location	Number (mean FU years)	Results (previous studies)<br><br>Effect of baseline HDL-C on cancer risk (adjusted analysis)	Results (current study)<br><br>Effect of longitudinal HDL-C change on cancer risk (adjusted analysis)
**Stomach (C16)**					
Current study	Cohort	Korea	2.8 million (8)	Normal HDL-C v. low HDL-C<br>0.96 (0.93-0.98) (total)<br>0.98 (0.94-1.01) (men)<br>0.98 (0.95-1.02) (women)	Overall aHR (95% CI) (Reference: persistent normal HDL-C)<br>1.05 (1.02, 1.09) in persistent low HDL-C<br>1.05 (1.02, 1.08) in normal-to-low HDL-C<br><br>Sub-analysis by sex<br>(men) 1.05 (1.01, 1.09) in normal-to-low HDL-C<br>(women) 1.11 (1.06, 1.16) in persistent low HDL-C<br>1.07 (1.02, 1.13) in low-to-normal<br>1.07 (1.02, 1.12) in normal-to-low<br><br>Sub-analysis by smoking<br>(never smokers)<br>1.07 (1.03, 1.12) in persistent low HDL-C<br>1.05 (1.00, 1.10) in low-to-normal<br>1.06 (1.02, 1.10) in normal-to-low<br>(smokers)<br>1.08 (1.03, 1.15) in persistent low<br>1.06 (1.02, 1.12) in normal-to-low<br><br>Subgroup analysis by age group<br>(age<65 years): no significant<br>(age≥65years)<br>1.07 (1.03, 1.12) in persistent low HDL-C<br>1.06 (1.02, 1.11) in normal-to-low HDL-C<br><br>Further increase of HDL-C among baseline normal group did not reduce or increase gastric cancer risk
					A decrement of absolute HDL-C level (ΔHDL-C < -10 mg/dL) increased the risk of stomach cancers: 1.05 (1.02, 1.08)<br>An increment of absolute HDL-C level (ΔHDL-C ≥ 25 mg/dL) increased the

					risk of stomach cancers: 1.07 (1.00, 1.14)
Ahn 2013	Cohort	Finland men	29,093 (18)	NS	
Nam 2018	Cohort	Korea	10,328 (10)	HR 2.67 (1.14-6.16) for low HDL-C comparing to normal HDL (men≥40, women≥50 mg/dL)	
Lim 2022	Cohort	Korea women	2.7 million (9)	(overall) NS<br>(Premenopausal) NS<br>(Postmenopausal) reference: Lowest quantile (<48)<br>HR 0.93 (0.89–0.98) for Q3<br>HR 0.90 (0.85–0.95) for Q4	
**Colorectal (C18-C20)**					
Current study	Cohort	Korea	2.8 million (8)	0.98 (0.95-1.00) (total)<br>1.00 (0.96-1.04) (men)<br>0.95 (0.92-0.98) (women)	Overall aHR (95% CI) (Reference: persistent normal HDL-C)<br>1.08 (1.05, 1.12) in normal-to-low HDL-C<br><br>Sub-analysis by sex<br>(men) 1.10 (1.05, 1.15) in normal-to-low HDL-C<br>(women) 1.06 (1.01, 1.10) in persistent low HDL-C<br>1.09 (1.04, 1.14) in normal-to-low<br><br>Sub-analysis by smoking<br>(never smokers)<br>1.05 (1.01, 1.09) in persistent low HDL-C<br>1.09 (1.05, 1.13) in normal-to-low<br>(smokers)<br>1.11 (1.05, 1.17) in normal-to-low<br><br>Subgroup analysis by age group<br>(age<65 years)<br>1.06 (1.01, 1.12) in persistent low HDL-C<br>1.06 (1.01, 1.12) in normal-to-low HDL-C<br>(age≥65years)<br>1.09 (1.05, 1.14) in normal-to-low HDL-C<br><br>Further increase of HDL-C (Δ HDL-C ≥15mg/dL) among baseline normal

					group reduce colorectal cancer risk: 0.94 (0.9, 0.99)
					A decrement of absolute HDL-C level (ΔHDL-C < -10 mg/dL) increased the risk of colorectal cancers: 1.07 (1.04, 1.11)<br>An increment of absolute HDL-C level reduced the risk of colorectal cancers:<br>0.96 (0.93, 0.99) (ΔHDL-C :5-15 mg/dL)<br>0.94 (0.90, 0.98) (ΔHDL-C : 15-25 mg/dL)
Duijnhoven 2011	Cohort	Europe	521,448	Colon cancer: inverse association<br>Rectal cancer: NS	
Ahn 2013	Cohort	Finland men	29,093 (18)	NS	
Fang Z 2021	Cohort	UK	380,087 (10.3)	NS	
Chandler 2016	Cohort	US women	15,602 (19)	HR 0.63 (highest vs. lowest quartile)	
Bowers K 2006	Cohort	Finland men	29,133	NS	
**Liver (C22)**					
Current study	Cohort	Korea	2.8 million (8)	Normal HDL-C v. low HDL-C<br>0.98 (0.95-1.02) (total)<br>0.93 (0.88-0.97) (men)<br>1.07 (1.00-1.14) (women)	Overall aHR (95% CI) (Reference: persistent normal HDL-C)<br>1.17 (1.12, 1.23) in persistent low HDL-C<br>1.07 (1.02, 1.12) in low-to-normal HDL-C<br>1.26 (1.21, 1.32) in normal-to-low HDL-C<br><br>Sub-analysis by sex<br>(men) 1.24 (1.18, 1.30) in normal-to-low HDL-C<br>(women) 1.08 (1.01, 1.15) in persistent low HDL-C<br>1.18 (1.09, 1.26) in normal-to-low<br><br>Sub-analysis by smoking<br>(never smokers)<br>1.17 (1.11, 1.24) in normal-to-low<br>(smokers)<br>1.13 (1.05, 1.22) in persistent low

					1.30 (1.22, 1.38) in normal-to-low<br><br>Subgroup analysis by age group<br>(age<65 years)<br>1.21 (1.12, 1.30) in persistent low HDL-C<br>1.32 (1.23, 1.41) in normal-to-low HDL-C<br>(age≥65years)<br>1.14 (1.07, 1.21) in persistent low HDL-C<br>1.09 (1.02, 1.17) in low-to-normal HDL-C<br>1.22 (1.16, 1.29) in normal-to-low HDL-C<br><br>Further increase of HDL-C (Δ HDL-C ≥15mg/dL) among baseline normal group increased liver cancer risk: 1.15 (1.08, 1.21)
					A decrement of absolute HDL-C level (ΔHDL-C < -10 mg/dL) increased the risk of liver cancers: 1.28 (1.24, 1.34)<br>An increment of absolute HDL-C level increased the risk of liver cancers:<br>1.14 (1.07, 1.21) (ΔHDL-C : 15-25 mg/dL)<br>1.41 (1.3, 1.53) (ΔHDL-C ≥ 25 mg/dL)
Ahn 2013	Cohort	Finland men	29,093 (18)	RR 0.61 (0.38-0.97) (highest vs. lowest quartile)	
Nderitu 2017	Cohort	Sweden	509,436 (13.6)	HR 2.40 (1.47–3.92) (lowest vs. Highest quartile)	
Pederson 2020	Cohort	Denmark	107,323 (liver-biliary cancer 150)	No association	
**Gallbladder-biliary (C23,C24)**					
Current study	Cohort	Korea	2.8 million (8)	Normal HDL-C v. low HDL-C<br>0.99 (0.94-1.04) (total)<br>1.00 (0.93-1.08) (men)<br>0.98 (0.92-1.05) (women)	Overall aHR (95% CI) (Reference: persistent normal HDL-C)<br>C23: 1.12 (1.02, 1.23) in low-to-normal HDL-C<br>C24:<br>1.02 (1.00, 1.05) in persistent low HDL-C<br>0.97 (0.95, 1.00) in low-to-normal<br>1.11 (1.08, 1.13) in normal-to-low<br><br>Sub-analysis by sex<br>C23, C24: no significance

					Subgroup analysis by age group<br>C23<br>(age<65 years) not significant<br>(age≥65years) 1.12 (1.01, 1.25) in normal-to low HDL-C<br>C24<br>(age<65 years) 1.19 (1.04, 1.36) in normal-to low HDL-C<br>(age≥65years) not significant
					A decrement of absolute HDL-C level (ΔHDL-C < -10 mg/dL) increased the risk of gallbladder and bilairy cancer<br>(C23) : 1.04 (0.95, 1.15)<br>(C24) : 1.09 (1.02, 1.17)<br><br>Further increase of HDL-C among baseline normal group did not reduce or increase gallbladder and biliary cancer risk.
Andreotti 2007	Case-control	China	858 control. 264 GB cancer, 141 bile duct cancer	Gallbladder, bile duct cancer was inverse associated with HDL-C (only age, sex adjusted)	
**Pancreas (C25)**					
Current study	Cohort	Korea	2.8 million (8)	Normal HDL-C v. low HDL-C<br>0.92 (0.88-0.97) (total)<br>0.92 (0.85-0.99) (men)<br>0.91 (0.85-0.97) (women)	Overall<br>1.11 (1.05, 1.17) in normal-to-low<br><br>Sub-analysis by sex<br>(men) 1.12 (1.02, 1.23) in low-to-normal HDL-C<br>1.10 (1.01, 1.19) in normal-to-low HDL-C<br>(women) 1.08 (1.01, 1.15) in persistent low HDL-C<br>1.12 (1.03, 1.20) in normal-to-low<br><br>Subgroup analysis by age group<br>(age<65 years)<br>1.11 (1.01, 1.23) in persistent low HDL-C<br>1.11 (1.00, 1.23) in low-to-normal HDL-C<br>1.21 (1.10, 1.32) in normal-to-low HDL-C<br>(age≥65years)<br>Not significant

					Further increase of HDL-C among baseline normal group did not reduce or increase pancreatic cancer risk.
					A decrement of absolute HDL-C level (ΔHDL-C < -10 mg/dL) increased the risk of pancreatic cancer: 1.07 (1.02, 1.13)
Ahn 2013	Cohort	Finland men	29,093 (18)	NS	
Meinhold 2008	Cohort	USA	27,035 (16.1)	NS	

NS, non-significance. ΔHDL-C = (HDL-C at follow-up) - (HDL-C at baseline).
