# Supplementary material for: A population-based cohort study of longitudinal change of high-density lipoprotein cholesterol impact on gastrointestinal cancer risk
Source: Nat Commun. 2024 Apr 4;15:2923. doi: 10.1038/s41467-024-47193-9 (PMC10994902; doi:10.1038/s41467-024-47193-9)
Supplement: Supplementary file 3 — Reporting Summary [file 41467_2024_47193_MOESM3_ESM.pdf]

nature portfolio

Corresponding author(s): Su Youn Nam

Last updated by author(s): Jan 25, 2024

Reporting Summary

Nature Portfolio wishes to improve the reproducibility of the work that we publish. This form provides structure for consistency and transparency in reporting. For further information on Nature Portfolio policies, see our Editorial Policies and the Editorial Policy Checklist.

Statistics

For all statistical analyses, confirm that the following items are present in the figure legend, table legend, main text, or Methods section.

n/a	Confirmed	
☐	☒	The exact sample size (n) for each experimental group/condition, given as a discrete number and unit of measurement
☐	☒	A statement on whether measurements were taken from distinct samples or whether the same sample was measured repeatedly
☐	☒	The statistical test(s) used AND whether they are one- or two-sided<br>*Only common tests should be described solely by name; describe more complex techniques in the Methods section.*
☐	☒	A description of all covariates tested
☐	☒	A description of any assumptions or corrections, such as tests of normality and adjustment for multiple comparisons
☐	☒	A full description of the statistical parameters including central tendency (e.g. means) or other basic estimates (e.g. regression coefficient) AND variation (e.g. standard deviation) or associated estimates of uncertainty (e.g. confidence intervals)
☐	☒	For null hypothesis testing, the test statistic (e.g. F, t, r) with confidence intervals, effect sizes, degrees of freedom and P value noted<br>*Give P values as exact values whenever suitable.*
☐	☐	For Bayesian analysis, information on the choice of priors and Markov chain Monte Carlo settings
☐	☒	For hierarchical and complex designs, identification of the appropriate level for tests and full reporting of outcomes
☐	☒	Estimates of effect sizes (e.g. Cohen's d, Pearson's r), indicating how they were calculated

Our web collection on statistics for biologists contains articles on many of the points above.

Software and code

Policy information about availability of computer code

Data collection

In this large population-based cohort study, we used data from the Korean NHISS (REQ0000045293), which cover approximately 98% of the Korean population aged ≥ 40 years every two years. We gathered the Base One Foundation Component Library (BFC), T20, the National General Health Examination (NGHE), and national cancer screening from NHISS.

We extracted sex and economic status from BFC; the disease codes of the International Classification of Disease, 10th revision (ICD 10) from T20 disease code; and demographic and laboratory data from the NGHE. The questionnaires included chronic diseases such as diabetes mellitus, hypertension, cerebrovascular disease, and ischemic heart disease, medication (anti-hypertensive drug, ant-diabetes drug, drug for heart disease, antiplatelet or anticoagulant drug, lipid lowering drug, etc), smoking status, alcohol consumption frequency, physical activity (frequency per week), and family history. Questionnaires also included liver-related variables (any liver disease history, chronic hepatitis B, chronic hepatitis C, and liver cirrhosis). Laboratory data included body mass index (BMI, weight/height2 [kg/m2]), blood pressure, lipid and fasting glucose levels. Weight and height were directly measured on the day of the health examination and blood samples were collected after 12 h of fasting. Fasting serum glucose and lipid levels were measured in units of milligram/deciliter (mg/dL) using fresh serum in each screening center. Age was defined as the age at the time of national general health examination. Economic status was extracted as household income twentile. Smoking status was categorized with never, past, or current smoker. Alcohol consumption frequency was classified as none, 1/week, 2-3/week, 4-5/week, and ≥ 6/week. Moderate physical activity refers to “walking or exercising and feeling mild dyspnea for more than 30 min per day.”

Data analysis

All analyses were performed using the SAS software (version 9.4; SAS Institute, Cary, NC, USA).

Statistics for the variables are presented as numbers (percentages) for categorical variables and as means (standard deviations) or medians (interquartile ranges) for continuous variables. We calculated person-years from baseline age to the first date of diagnosis of any cancer, death, or December 31, 2017. HDL-C levels were extracted at baseline (2009) and follow-up (2013), and changes in HDL-C levels were

classified into four groups: persistent normal (normal-to-normal), change from normal to low (normal-to-low), change from low to normal (low-to-normal), and persistent low (low-to-low) (Figure 1B and Supplementary p 5). According to the Adult Treatment Panel III (ATP III),15 normal HDL-C levels were defined as HDL-C ≥ 40 mg/dL in men and ≥50 mg/dL in women. Low HDL-C levels were defined as HDL-C < 40 mg/dL in men and < 50 mg/dL in women. Cancer risk according to HDL-C change was measured with hazard ratios (HRs) and 95% confidence intervals (CIs) using Cox proportional regression analysis. The persistently normal group was set as the reference group. The association between covariates and cancer risk was assessed using the Cox regression analysis. We performed a multivariate analysis adjusted for significant confounders, such as age, sex, BMI, smoking status, alcohol intake, physical activity, economic status, use of lipid lowering drugs, hypertension, diabetes, cerebrovascular disease, heart disease, and physical activity. Liver cancer analysis was additionally adjusted for liver factors, such as liver disease, chronic hepatitis B, chronic hepatitis C, liver cirrhosis, and triglyceride level. A directed acyclic graph provides an assumed causal framework for covariate adjustments (Supplementary Fig. 1). Among the final eligible population, the highest missing rate was for economic status (1.7%). Therefore, we deleted missing data list-wise in the adjusted analysis (Supplementary p3).

To assess the cancer risk by HDL-C change among the baseline normal group, the persistent normal group was further classified into persistent normal and normal-increase (baseline normal and increase ≥15 mg/dL at follow-up from baseline) groups (Fig. 1B). To conduct sensitivity analysis for the impact of exposure duration, we excluded cancers that developed within 1 year from the second measurement of HDL-C level and then analyzed the impact of HDL-C change on each cancer risk (model II). We conducted an interaction analysis (joint test) between the well-known important cofactors (sex and smoking status) and HDL-C level changes in the cancer risk. And then, a subgroup analysis was performed based on sex and smoking status. We also classified HDL-C change using continuous value. Change of HDL-C [ΔHDL-C = (HDL-C at follow-up) - (HDL-C baseline)] were classified as decrease (ΔHDL-C < -10 mg/dL, ΔHDL-C= -5 ~ -10 mg/dL), stable (ΔHDL-C = -5 ~ 5 mg/dL), increase (ΔHDL-C=5 ~ 15 mg/dL, ΔHDL-C = 15 ~ 25 mg/dL, and ΔHDL-C ≥ 25 mg/dL).

For manuscripts utilizing custom algorithms or software that are central to the research but not yet described in published literature, software must be made available to editors and reviewers. We strongly encourage code deposition in a community repository (e.g. GitHub). See the Nature Portfolio guidelines for submitting code & software for further information.

Data

Policy information about availability of data

All manuscripts must include a data availability statement. This statement should provide the following information, where applicable:

- Accession codes, unique identifiers, or web links for publicly available datasets
- A description of any restrictions on data availability
- For clinical datasets or third party data, please ensure that the statement adheres to our policy

Raw data are not publicly available because analysis can be conducted only in a closed office regulated by National Health Insurance Service System (NHISS). If someone want to use the raw data, they have to request to NHISS. After NHISS approve the use of data, they can analyze them in a closed office regulated by NHISS. https://nhiss.nhis.or.kr/bd/ay/bdaya001iv.do

Research involving human participants, their data, or biological material

Policy information about studies with human participants or human data. See also policy information about sex, gender (identity/presentation), and sexual orientation and race, ethnicity and racism.

Reporting on sex and gender

Sex is biological sex in this study.
We conducted interaction analysis for the link of sex and HDL-C change in the risk of each cancer.
And we performed subgroup analysis according to sex.

Reporting on race, ethnicity, or other socially relevant groupings

We did not describe race because most people covered by NHISS are Korean and NHISS does not provide race informations.

Population characteristics

In 2010, 6.18 million underwent both national heath examination and any cancer screening. Cancers before 2010 were excluded and subjects who did not undergo gastric cancer screening. A total of 4.373 million underwent both national heath examination and gastric cancer screening. After the exclusion of subjects with any cancer diagnosed within 1year and those who died within 1year, 4.323 million persons were enrolled at baseline. After excluding non-participants for the national general health examination or absence of HDL-C value in 2014, 3.135 million individuals were eligible. After further excluding unknown sex type, 3,130,795 persons (1,387,648 men [44%] and mean age of 54 years) are eligible [model I] and followed up until 2021 (Fig. 1A). The baseline characteristics of the included and excluded individuals was provided in Supplementary Table 1.

Recruitment

Among 6.18 million who underwent both NGHE and any cancer screening from January to December 2010, patients with any pre-existing cancer and persons who did not undergo gastric cancer screening were excluded. Among cancer free individuals who underwent both NGHE and National gastric cancer screening from January to December 2010 (4.373million), we excluded persons with any C-codes or death within 12 months from index month (the month of health examination) (n=49,991) (Fig. 1A). Death data from the National Statistical Office were also provided by the National Health Insurance Service. Nearly complete sensing of new cancer is possible through extracting C code form NHISS. In order to receive a special exemption for cancer, the cancer code should be registered with diagnostic evidence of cancer such as pathologic results at hospital. If any cancer code is registered, the patients pay just 5% for cancer related medical services. Therefore, cancer (C) code extracted from NHISS is highly reliable. Individuals who did not undergo NGHE in 2014 or absence of HDL-C values in 2014 (n= 1,188,176) and unknown sex type (n=3991) were excluded [model I; n=3,130,795, Fig 1B]. All cancer codes (C codes) were extracted up to December 2021. Common gastrointestinal cancers included gastric (C16), liver (C22), colorectal (C18, C19, C20), pancreatic (C25), gallbladder (C23), and biliary (C24) cancers.

Ethics oversight

This study was approved by the Institutional Review Board of Kyungpook National University Hospital, Chilgok (KNUHC 2017-12-022).

Note that full information on the approval of the study protocol must also be provided in the manuscript.

Field-specific reporting

Please select the one below that is the best fit for your research. If you are not sure, read the appropriate sections before making your selection.

- [ ] Life sciences
- [x] Behavioural & social sciences
- [ ] Ecological, evolutionary & environmental sciences

For a reference copy of the document with all sections, see nature.com/documents/nr-reporting-summary-flat.pdf

Behavioural & social sciences study design

All studies must disclose on these points even when the disclosure is negative.

Study description	cohort study
Research sample	In this large population-based cohort study, we used data from the Korean NHISS (REQ202204452-004), which cover approximately 98% of the Korean population aged ≥ 40 years every two years. We gathered the Base One Foundation Component Library (BFC), T20, the National General Health Examination (NGHE), and national cancer screening from National Health Insurance Service system (NHISS).
Sampling strategy	Subjects who underwent a national general health examination and cancer screening in 2010 and national general health examination in 2014 were included in this study. Patients who had been diagnosed with cancer before 2010 and within 1 year of baseline enrollment, and those who died within 1 year of enrollment were excluded (Fig. 1A). Individuals who did not undergo a national general health examination in 2014 were also excluded. All cancer codes (C codes) were extracted up to December 2021.
Data collection	We used NHISS data
Timing	Subjects who underwent a national general health examination and cancer screening in 2010 and national general health examination in 2014 were included in this study.
Data exclusions	Patients who had been diagnosed with cancer before 2010 and within 1 year of baseline enrollment, and those who died within 1 year of enrollment were excluded (Fig. 1A). Individuals who did not undergo a national general health examination in 2014 were also excluded.
Non-participation	NA
Randomization	NA

Reporting for specific materials, systems and methods

We require information from authors about some types of materials, experimental systems and methods used in many studies. Here, indicate whether each material, system or method listed is relevant to your study. If you are not sure if a list item applies to your research, read the appropriate section before selecting a response.

Materials & experimental systems

n/a	Involved in the study	
x		Antibodies
x		Eukaryotic cell lines
x		Palaeontology and archaeology
x		Animals and other organisms
	x	Clinical data
x		Dual use research of concern
x		Plants

Methods

n/a	Involved in the study	
x		ChIP-seq
x		Flow cytometry
x		MRI-based neuroimaging

Clinical data

Policy information about clinical studies

All manuscripts should comply with the ICMJEguidelines for publication of clinical research and a completedCONSORT checklist must be included with all submissions.

Clinical trial registration	This is not clinical trial. National data based epidemiological study.
Study protocol	(REQ202204452-004)
Data collection	We used National health examination data (REQ202204452-004)
Outcomes	cancer risk
